# Supplementary material for: Evaluation of Electrospun Self-Supporting Paper-Like Fibrous Membranes as Oil Sorbents
Source: Membranes (Basel). 2021 Jul 8;11(7):515. doi: 10.3390/membranes11070515 (PMC8306189; doi:10.3390/membranes11070515)
Supplement: Supplementary file 1 [file membranes-11-00515-s001.zip › membranes-1288953-supplementary.pdf]

membranes

MDPI

Supplementary Information

Evaluation of Electrospun Self-Supporting Paper-Like Fibrous Membranes as Oil Sorbents

Adele Folino [1], Claudia Triolo [2], Beatrix Petrovičová [2], Fabiolo Pantò [3], Demetrio A. Zema [1,*] and Saveria Santangelo [2,*]

1 Department of Agriculture, Mediterranean University of Reggio Calabria, Località Feo di Vito, I-89122 Reggio Calabria, Italy; adelefolino@alice.it
2 Department of Civil, Energy, Environmental and Materials Engineering. (DICEAM), Mediterranean University, Località Feo di Vito, I-89122 Reggio Calabria, Italy; claudia.triolo@unirc.it (C.T.); beatrix.petrovicova@unirc.it (B.P.)
3 Institute of Advanced Technologies for Energy (ITAE), Italian National Research Council (CNR), I-98126 Messina, Italy; fabiola.panto@itae.cnr.it
* Correspondence: dzema@unirc.it (D.A.Z.); saveria.santangelo@unirc.it (S.S.)

Citation: Folino, A.; Triolo, C.; Petrovičová, B.; Pantò, F.; Zema, D.A.; Santangelo, S. Evaluation of Electrospun Self-Supporting Paper-Like Fibrous Membranes as Oil Sorbents. *Membranes* **2021**, *11*, 515. https://doi.org/10.3390/membranes11070515

Academic Editor: Scott M. Husson

Received: 22 June 2021
Accepted: 6 July 2021
Published: 8 July 2021

Publisher's Note: MDPI stays neutral with regard to jurisdictional claims in published maps and institutional affiliations.

CC BY

Copyright: © 2021 by the authors. Licensee MDPI, Basel, Switzerland. This article is an open access article distributed under the terms and conditions of the Creative Commons Attribution (CC BY) license (http://creativecommons.org/licenses/by/4.0/).

Table S1. Main characteristics and results reported in the studies on PAN membranes used as oil sorbents.

Ref.	ENM type	Spinnable Solution	Spinning Condition	Heat Treatment	NF Diameter and SSA	Adsorption Method	Oil Type	Oil Recovery [g g^{-1}]
[1]	MCNFF	0.8g PAN + 10g DMF	1.5 mL/h<br>25 kV<br>15 cm	280 °C/2h/air<br>800 °C/2h/Ar	n.a.<br>16.0 m^2 g^{-1}	Immersion	Ethanol<br>Pump oil<br>Mineral oil<br>Silicone oil<br>Corn oil	17.5<br>37.0<br>27.7<br>78.5<br>42.8
		0.8g PAN + 10g DMF + 0.5g PTA			280 nm<br>20.9 m^2 g^{-1}	Immersion	Ethanol<br>Pump oil<br>Mineral oil<br>Silicone oil<br>Corn oil<br>Petroleum/water mixture	62.6<br>73.8<br>64.0<br>138.4<br>94.0<br>100
						Filter separation	Oil-water mixture	100
[2]	PAN–silica NFs	7% PAN+ 15:1 DMF:AA + 0.5% TEOS	0.54-0.6 mL/h<br>0.6-0.8 kV/cm	280 °C/2h/air<br>900 °C/2h/N_2	200-300 nm<br>41.8 m^2 g^{-1}	Gravity separation	Pump oil<br>Silicon oil<br>Olive oil<br>Bean oil/water<br>Sunflower seed oil/water	60*<br>100*<br>98*<br>30*<br>22*
[3]	SiO_2@MUF/PAN	10% 3:1 PAN: MUF + 100 g DMF	1 mL/h<br>20 kV<br>15 cm	110 °C/2h/air	120 nm<br>20.89 m^2 g^{-1}	Immersion	Engine oil<br>Pump oil<br>Diesel<br>Peanut oil	21*<br>16*<br>11*<br>15*
		10% 3:1 PAN: MUF + 100 g DMF + 0.45% SiO_2			110 nm<br>21.47 m^2 g^{-1}		Engine oil<br>Pump oil<br>Diesel<br>Peanut oil	22*<br>17*<br>13*<br>19*
		10% 3:1 PAN: MUF + 100 g DMF + 0.90% SiO_2			149 nm<br>23.45 m^2 g^{-1}		Engine oil<br>Pump oil<br>Diesel<br>Peanut oil	22.7*<br>18.5*<br>13.1*<br>19.1*
		10% 3:1 PAN: MUF + 100 g DMF + 1.80% SiO_2			137 nm<br>7.74 m^2 g^{-1}		Engine oil<br>Pump oil<br>Diesel<br>Peanut oil	14.5*<br>14.5*<br>9.5*<br>17*

*estimated data.

Syntheses

The procedure followed for the synthesis of the sorbent membranes is sketched in Figure S1. The spinnable solution to produce PAN-based fibrous membranes was prepared by dissolving 6.5 wt% PAN in DMF. PAN/PMMA/DMF solutions with different PAN:PMMA mass ratios were also prepared, keeping total content of the polymer constant. PMMA acted as a sacrificial pore-forming agent. Electrospinning was carried out in air by using 20-mL syringe with a 4-mm long and 0.8-mm gauge stainless steel needle. The PAN/DMF and PAN/PMMA/DMF solutions were fed at volumetric rates of 1.41 mL h^{-1} and 1.00 mL h^{-1}, respectively. In the former case, applied voltage was 17.2 kV; in the latter, it was 15.0 kV. Collection distance was kept constant at 11 cm. The strong jet elongation made most DMF to evaporate during the electrospinning [4]; the residual solvent was removed via drying over night at room temperature. Then, the as-spun PAN and PAN/PMMA membranes were peeled-off from the collector and thermally treated.

Stabilisation was carried in static air under the same conditions for all the investigated membranes; carbonization was operated at flow of 100 cc min^{-1} N_2. The pure PAN membranes were carbonized at different temperatures (500, 650 or 800 °C) in order to evaluate the effect of the carbonization temperature (T_C) on their microstructure, mechanical robustness and adsorption efficiency. For the membranes derived from the blends PAN/PMMA, T_C was set at 650 °C. At this temperature, pure PAN membrane showed water repellence and the highest oil recovery (see main text).

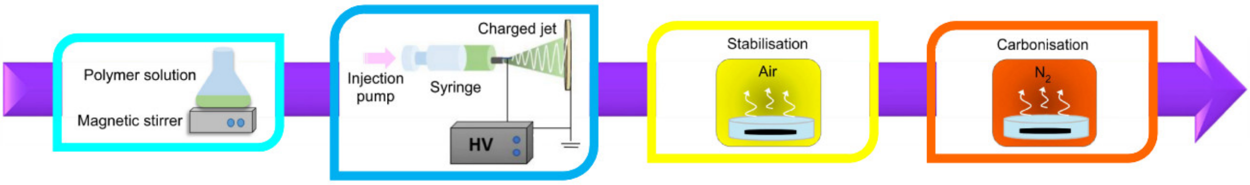

Figure S1. Sketch of the procedure followed to for the synthesis of the sorbent membranes.

Setup for contact angle measurements

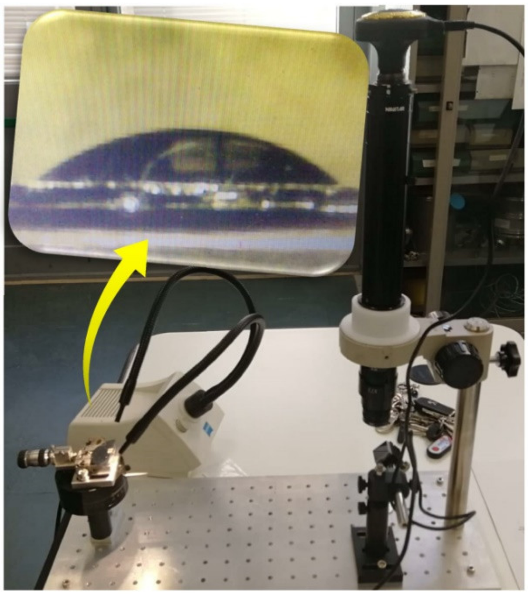

Figure S2. Experimental setup to measure the contact angle of a water droplet on surface of the produced membranes. A sample-holder, mounted on a rotating mechanical support, was used. A micrometer movement system finely controlled the position of the droplet, released via a calibrated syringe. A CCD camera mounted on an optical microscope, with a long focal distance, acquired the optical images of the droplets. Inset: image of a droplet caught at the optical microscope.

Adsorption experiments and tested liquids

Table S2. Main physical properties of the tested liquids.

Liquid	Mass density (g cm^{-3})	Ref.	Viscosity coefficient (mPa·s)	Ref.	Surface tension (mN/m)	Ref.
Water	1.000		0.002		72.8	
Olive oil	0.908	[5]	74.1	[5]	31.9	[5]
Sunflower seed oil	0.917	[6]	48.8	[7]	34.0	[6]
Motor oil	0.880	[6]	162.3	[8]	31.7	[6]

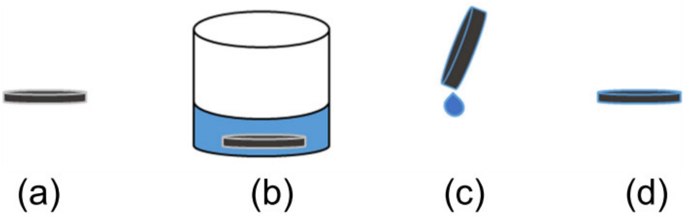

Figure S3. Sketch of the procedure followed to estimate the liquid recovery efficiency by immersion: after weighing, the membrane (**a**) is immersed in the liquid (**b**); after draining (c), the liquid-saturated membrane (**d**) is weighed.

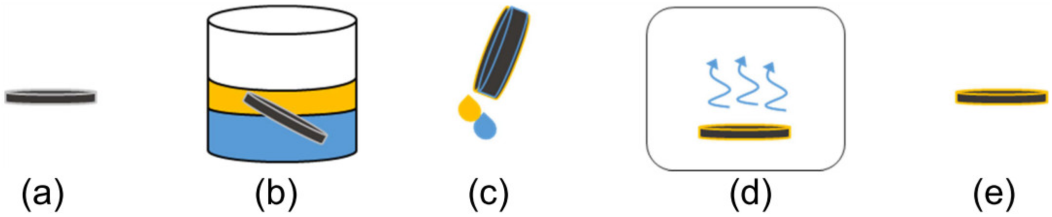

Figure S4. Sketch of the procedure followed to estimate the recovery efficiency in the case of the bi-phasic liquid: after weighing, the membrane (**a**) is immersed in the mixture oil/water (**b**); after draining (**c**), the liquid-saturated membrane is transferred in oven to remove water (**d**) and finally weighed (**e**).

Effect of thermal treatments

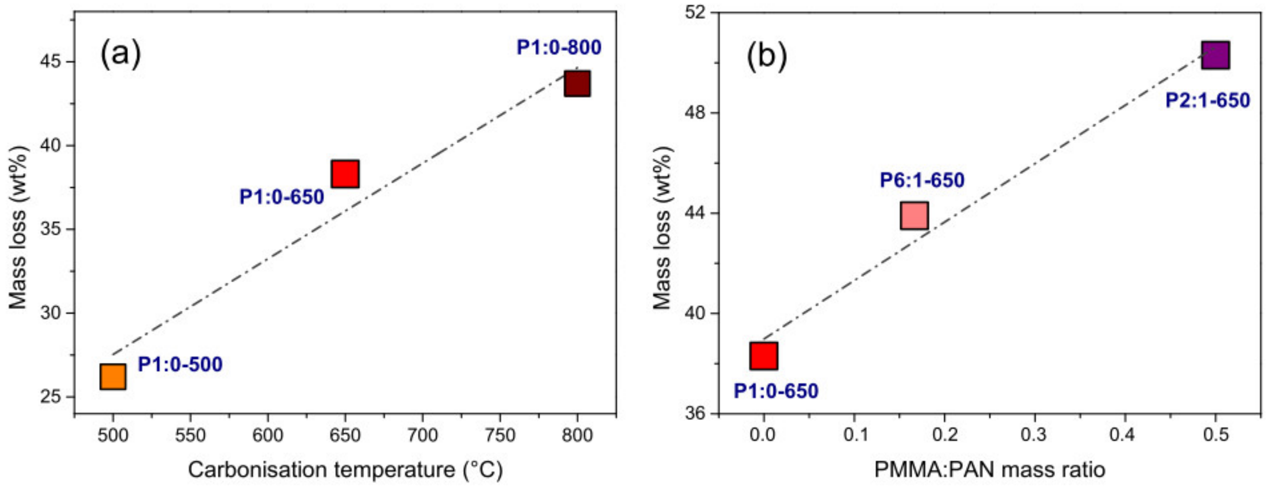

Figure S5. Mass loss during carbonisation of (**a**) PAN-based membranes at different temperatures and (**b**) PAN/PMMA-derived membranes with different PMMA:PAN ratios at 650 °C.

Membrane nanostructure

Table S3. Results of MRS and XRD analyses. MRS: D/G intensity ratio (I_D/I_G), calculated band amplitude ratio, as in the case of large disorder, and resulting average size of the graphite-like domains (L_C). XRD: angular position of the (002) reflection peak (θ_{002}) and corresponding interplanar spacing, calculated, using the Bragg's law, as $d_{002} = \lambda/2\sin\theta$, where λ (0.15451 nm) is the wavelength of $Cu\text{-}K_\alpha$ radiation source and θ_{002} is the diffraction angle; its decrease is indicative of the diminishing of heteroatoms intercalated between planes in the graphite-like domains.

**Membrane**	**MRS**			**XRD**	
	I_D/I_G	L_C **(nm)**	ω_G **(cm⁻¹)**	θ_{002} **(deg)**	d_{002} **(nm)**
P1:0-500	1.60 ± 0.02	2.75 ± 0.03	1578.4 ± 0.4	24.1 ± 0.4	0.37 ± 0.01
P1:0-650	1.55 ± 0.02	2.84 ± 0.04	1581.0 ± 0.5	24.6 ± 0.4	0.36 ± 0.01
P1:0-800	1.52 ± 0.03	2.89 ± 0.05	1587.9 ± 0.6	25.2 ± 0.4	0.35 ± 0.01
P6:1-650	1.52 ± 0.02	2.89 ± 0.04	1584.7 ± 0.5		
P2:1-650	1.46 ± 0.02	3.01 ± 0.04	1590.6 ± 0.5		

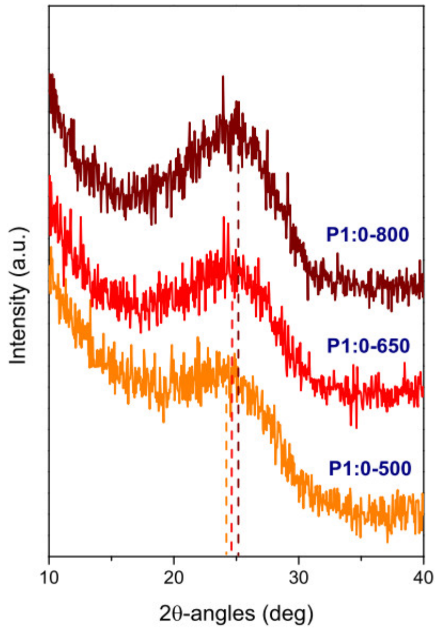

Figure S6. XRD patterns of PAN-derived membranes carbonised at different temperatures.

Adsorption experiments

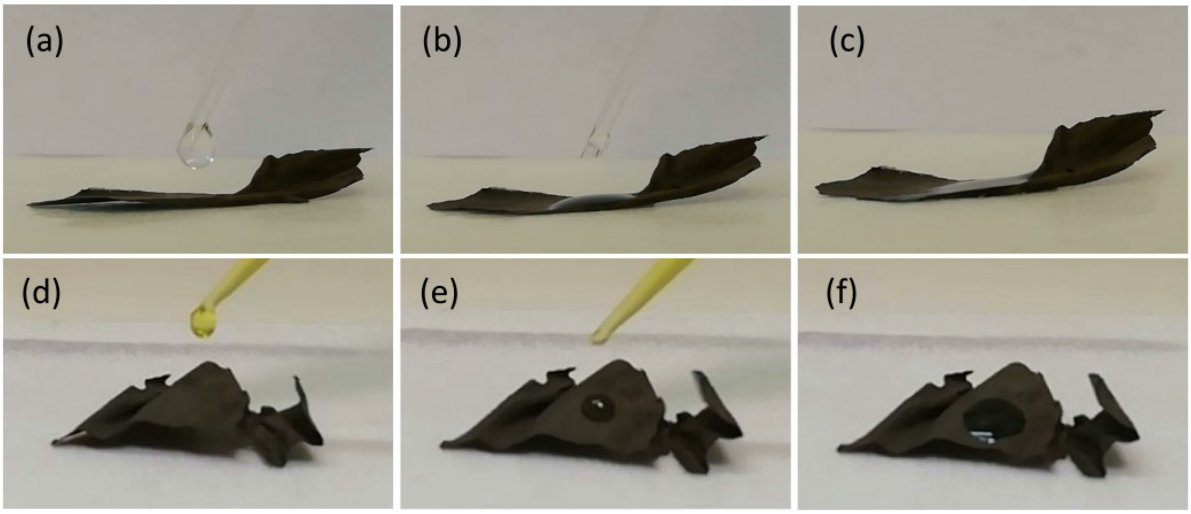

Figure S7. Spread of a drop of (**a**–**c**) water and (**d**–**f**) oil over membrane P1:0-500.

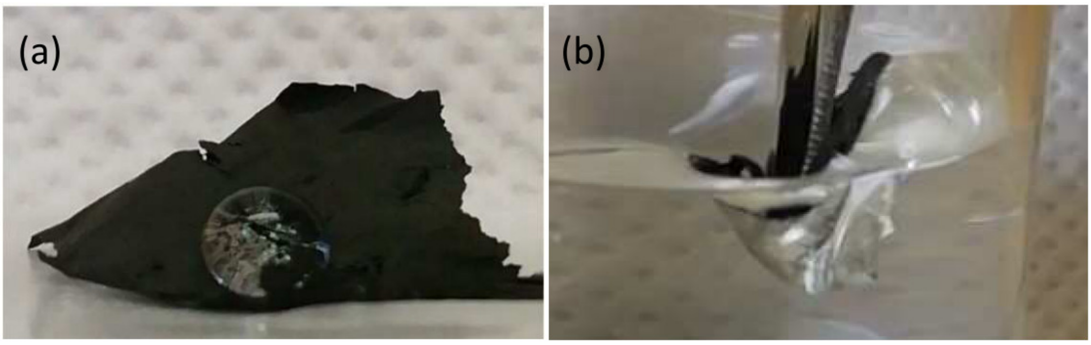

Figure S8. (**a**) A water drop over membrane P1:0-650. (**b**) Immersion of the membrane in water.

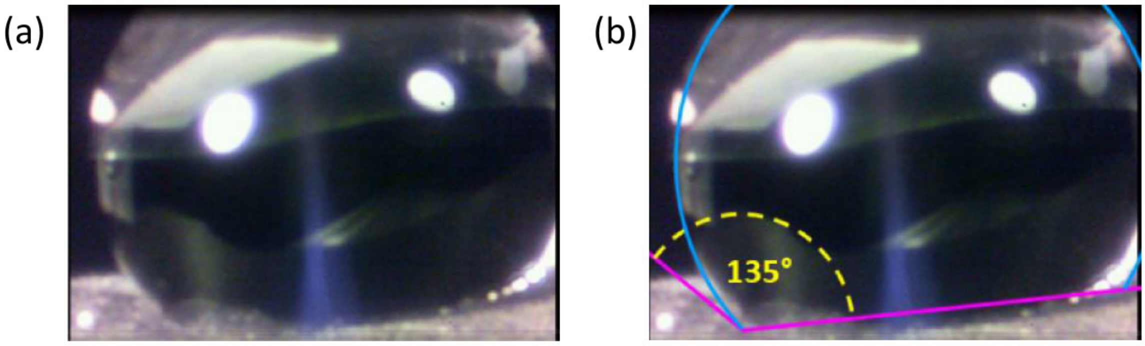

Figure S9. Water droplet contact angle measurement on membrane P1:0-650. (**a**) Droplet caught at the optical microscope; (**b**) scheme for the evaluation of the water contact angle.

Table S4. Recovery efficiencies measured in tests of immersion in water, olive oil (OO) and sunflower seed oil (SFO) and motor oil (MO).

Sample Code	Water (g g^{-1})	OO (g g^{-1})	SFO (g g^{-1})	MO (g g^{-1})
P1:0-500	78.9 ± 3.2	29.8 ± 1.2	94.8 ± 3.8	23.0 ± 1.0
P1:0-650	18.0 ± 0.8	61.0 ± 2.5	43.7 ± 1.8	67.8 ± 2.8
P1:0-800	11.5 ± 0.5	48.8 ± 2.0	53.6 ± 2.2	49.9 ± 2.0
P6:1-650	2.5 ± 0.1	4.3 ± 0.2	4.4 ± 0.2	14.7 ± 0.6
P2:1-650	87.6 ± 3.5	24.9 ± 1.0	21.6 ± 0.9	17.8 ± 0.8

Table S5. Water and oil recovery efficiencies (R_W and R_O) measured by immerging the sorbent membrane in 1:4 (v:v) oil/water mixture (spill tests) and corresponding oil/water selectivity ($S_{O/W}$).

Sample code	OO			SFO			MO		
	R_W (g g^{-1})	R_O (g g^{-1})	$S_{O/W}$	R_W (g g^{-1})	R_O (g g^{-1})	$S_{O/W}$	R_W (g g^{-1})	R_O (g g^{-1})	$S_{O/W}$
P1:0-500	2.3 ± 0.1	38.6 ± 1.6	16.8 ± 1.7	5.0 ± 0.2	38.4 ± 1.6	7.7 ± 0.7	3.2 ± 0.2	29.2 ± 1.2	9.1 ± 0.9
P1:0-650	26.3 ± 1.1	100.3 ± 4.1	3.8 ± 0.3	15.6 ± 0.7	89.0 ± 3.6	5.7 ± 0.5	43.2 ± 1.8	68.7 ± 2.8	1.6 ± 0.1
P1:0-800	3.7 ± 0.2	37.2 ± 1.5	10.1 ± 0.9	2.5 ± 0.1	32.2 ± 1.3	12.9 ± 1.3	8.1 ± 0.4	22.5 ± 0.9	2.8 ± 0.2
P6:1-650	0.3 ± 0.1	3.7 ± 0.2	12.6 ± 2.8	0.2 ± 0.1	5.5 ± 0.3	28.0 ± 8.0	0.2 ± 0.1	8.5 ± 0.4	35.5 ± 10.1
P2:1-650	1.0 ± 0.1	73.5 ± 3.0	73.5 ± 8.9	0.8 ± 0.1	54.8 ± 2.2	68.5 ± 9.0	0.8 ± 0.1	12.5 ± 0.5	15.6 ± 2.1

Correlations with properties of the tested oils

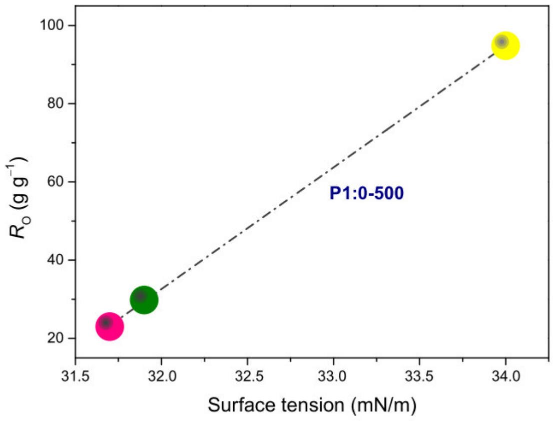

Figure S10. Relation between recovery efficiency and oil surface tension in adsorption tests of membrane P1:0-500.

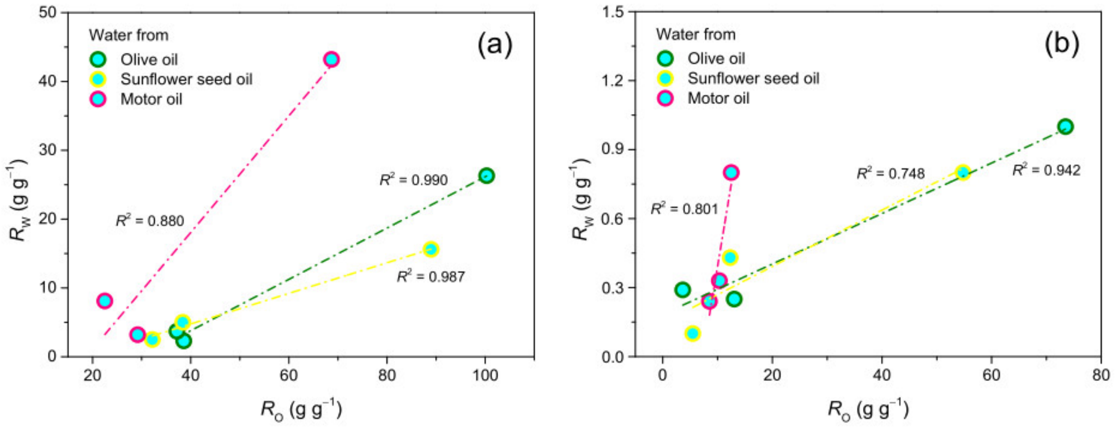

Figure S11. Plots of water- *vs* oil-recovery efficiency for (**a**) PAN-membranes carbonised at different temperatures and (**b**) PAN/PMMA-membranes with different (from 6:1 to 2:1) PAN:PMMA mass ratio carbonised at 650 °C.

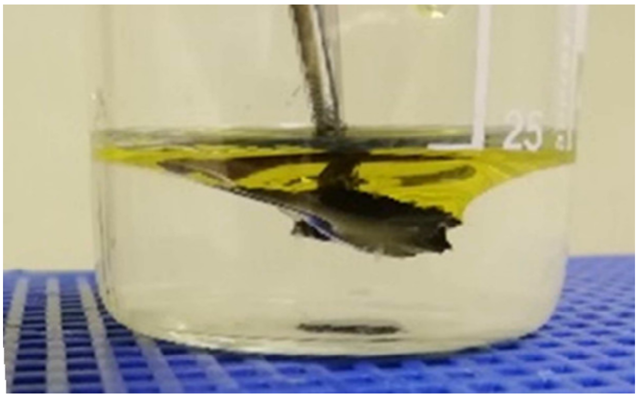

Figure S12. Image of the membrane immersion in the bi-phasic oil/water mixture in the spill tests.

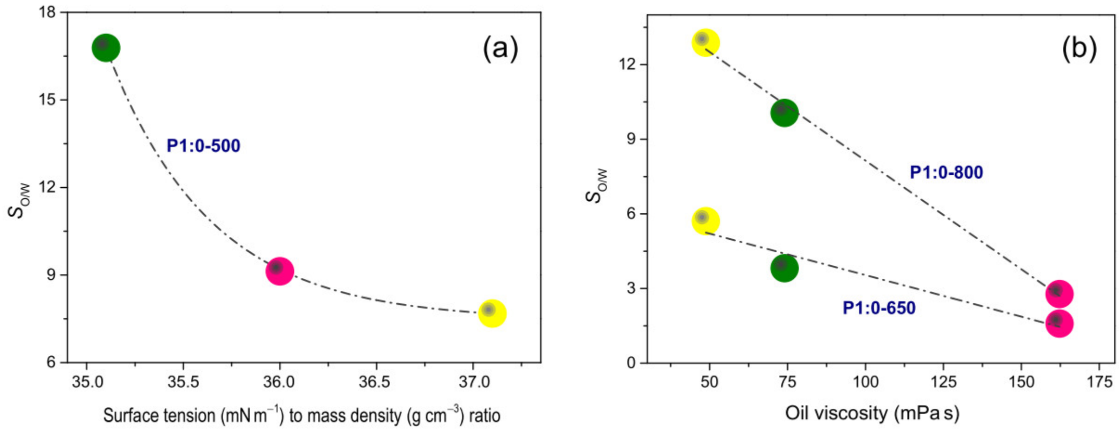

Figure S13. Dependence of the oil/water selectivity ($S_{O/W}$) of the PAN-derived membranes on the (**a**) strength of capillary action, as monitored by the oil surface tension to mass density ratio, and (**b**) oil viscosity.

References

1. Liu, H.; Cao, C.-Y.; Wei, F.-F.; Huang, P.-P.; Sun, Y.-B.; Jiang, L.; Song, W.-G. Flexible macroporous carbon nanofiber film with high oil adsorption capacity. *J. Mater. Chem. A* **2014**, *2*, 3557–3562.
2. Tai, M.H.; Tan, B.Y.L.; Juay, J.; Sun, D.D.; Leckie, J.O. A self-assembled superhydrophobic electrospun carbon-silica nanofiber sponge for selective removal and recovery of oils and organic solvents. *Chem. - A Eur. J.* **2015**, *21*, 5395–5402.
3. Yuan, J.; Gao, R.; Wang, Y.; Cao, W.; Yun, Y.; Dong, B.; Dou, J. A novel hydrophobic adsorbent of electrospun SiO2@MUF/PAN nanofibrous membrane and its adsorption behaviour for oil and organic solvents. *J. Mater. Sci.* **2018**, *53*, 16357–16370.

4. Bognitzki, M.; Czado, W.; Frese, T.; Schaper, A.; Hellwig, M.; Steinhart, M.; Greiner, A.; Wendorff, J.H. Nanostructured Fibers via Electrospinning. *Adv. Mater.* **2001**, *13*, 70–72.
5. Shin, C.; Chase, G.G. Water-in-Oil Coalescence in Micro-Nanofiber Composite Filters. *AIChE J.* **2004**, *50*, 343–350.
6. Esteban, B.; Riba, J.R.; Baquero, G.; Puig, R.; Rius, A. Characterization of the surface tension of vegetable oils to be used as fuel in diesel engines. *Fuel* **2012**, *102*, 231–238.
7. Diamante, L.M.; Lan, T. Absolute Viscosities of Vegetable Oils at Different Temperatures and Shear Rate Range of 64.5 to 4835 s −1. **2014**.
8. Qiao, Y.; Zhao, L.; Li, P.; Sun, H.; Li, S. Electrospun polystyrene/polyacrylonitrile fiber with high oil sorption capacity. *J. Reinf. Plast. Compos.* **2014**, *33*, 1849–1858.
